# Efficacy and safety of oral anticoagulants in the treatment of chronic kidney disease with atrial fibrillation or venous thromboembolism: a systematic review and meta-analysis

**DOI:** 10.3389/fphar.2025.1615284

**Published:** 2025-08-29

**Authors:** Qinan Yin, Xingyue Zheng, Xiaoqing Ni, Yin Wang, Xuefei Huang, Yujie Song, Lizhu Han, Youjin Huang, Yuan Bian

**Affiliations:** ^1^ Department of Pharmacy, Personalized Drug Research and Therapy Key Laboratory of Sichuan Province, Sichuan Provincial People’s Hospital, School of Medicine, University of Electronic Science and Technology of China, Chengdu, China; ^2^ Department of Pharmacy, Chengdu Women's and Children's Central Hospital, School of Medicine, University of Electronic Science and Technology of China, Chengdu, China; ^3^ Department of Vascular Surgery Center, Sichuan Academy of Medical Sciences and Sichuan Provincial People's Hospital, School of Medicine, University of Electronic Science and Technology of China, Chengdu, China

**Keywords:** venous thromboembolism, atrial fibrillation, chronic kidney disease, oral anticoagulation, systematic review

## Abstract

**Background:**

The choice of oral anticoagulants for patients with Chronic Kidney Disease (CKD) combined with venous thromboembolism (VTE) or atrial fibrillation (AF) remains controversial.

**Objective:**

To compare the efficacy and safety of warfarin and direct oral anticoagulants (DOACs) in the treatment of CKD with atrial fibrillation or venous thromboembolism.

**Methods:**

Relevant publications were sourced from databases like PubMed, Embase, Web of Science, Cochrane Library, and ClinicalTrials.gov up to 30 June 2024. Only RCTs assessing the efficacy and safety of warfarin and DOACs for treating CKD with AF or VTE were included in the meta-analysis. The review outcomes are thrombosis recurrence or VTE-related deaths and major bleeding for CKD patients with VTE, and stroke or systemic embolism and major bleeding for CKD patients with AF. The risk of bias in all included studies was evaluated using the Cochrane Collaboration’s tool.

**Results:**

After reviewing 540 studies, 15 randomized controlled trials (RCTs) with 16,361 participants were included. The study found that DOACs reduced the risk of hemorrhagic stroke compared to warfarin in patients with AF and CKD (RR = 0.455, 95% CI: 0.275–0.752, P = 0.002). There was no significant difference in ischemic stroke incidence between the two. DOACs also lowered the risk of major bleeding in patients with AF and CKD compared to warfarin (RR = 0.604, 95% CI: 0.442–0.825, P = 0.002), and significantly reduced the risk of intracranial bleeding (RR = 0.424, 95% CI: 0.287–0.626, P < 0.001). All five studies reported recurrent VTE or VTE-related deaths, showing no significant difference between warfarin and DOAC groups (RR = 0.663, 95% CI: 0.409–1.073, P = 0.094), Patients with renal dysfunction on either treatment had similar risks of major bleeding events (RR = 0.543, 95% CI: 0.209–1.407, P = 0.208).

**Conclusion:**

DOACs demonstrate superior efficacy and safety compared to warfarin in patients with AF and CKD. Additionally, DOACs exhibit comparable efficacy and safety to warfarin in patients with VTE and CKD.

**Systematic Review Registration:**

http://www.clinicaltrials.gov, identifier (CRD42024510727).

## Graphical Abstract

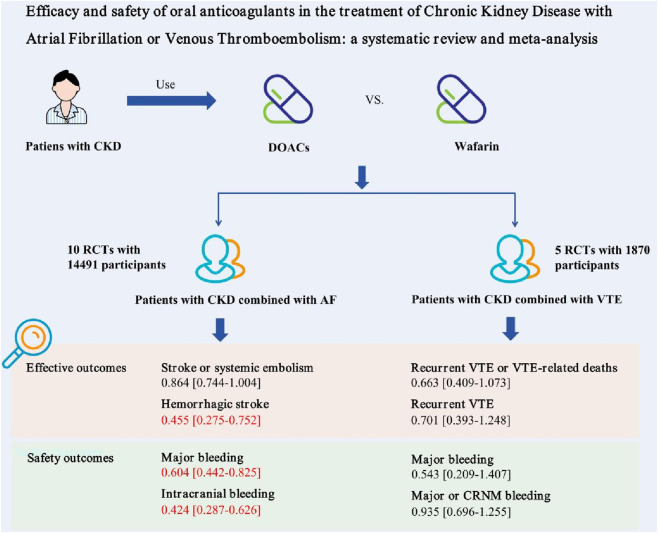

## 1 Introduction

Venous thromboembolism (VTE), encompasses deep vein thrombosis (DVT) and/or pulmonary embolism (PE), shedding of DVT can cause PE and is a manifestation of the same disease at different stages. It can severely affect patients’ quality of life and is the third leading cause of death globally (Klemen, 2020; Pastori, 2023; Yamashita et al., 2022). Anticoagulation is the main treatment for VTE patients (Stevens, 2021). Atrial fibrillation (AF) is a common persistent arrhythmia that worsens quality of life and raises the risk of death, stroke, heart failure and dementia. Anticoagulation significantly lowers stroke risk in AF patients (Joglar, 2024).

Chronic kidney disease (CKD) is identified by kidney damage (urinary albumin excretion rate ≥30 mg d^−1^ or equivalent) or decreased kidney function (eGFR <60 mL/min/1.73 m^2^) for 3 months or more (Inker, 2014). CKD is a pre-thrombotic condition that significantly raises the risk of arterial and VTE (Jones, 2024). AF is prevalent in this population, affecting 18% of CKD patients and 12%–25% of those with dialysis-dependent end-stage renal disease (ESKD). CKD heightens the risk of stroke, systemic embolism, heart failure, myocardial infarction, and overall mortality in AF patients (Ha et al., 2019).

Anticoagulation is an important measure to prevent AF stroke and treat VTE. However, due to the decreased renal function in patients with CKD, the metabolism and excretion capacity of the kidney are weakened, which limits the use of anticoagulant drugs and brings difficulties to the selection of drugs in clinical treatment. As one of the most commonly used oral anticoagulants, warfarin is metabolized by cytochrome P450 (CYP450) enzymes in the liver, independent of the kidney (only the excretion of inactive metabolites through the kidney), and current research evidence supports the safe and effective use of warfarin in mild to moderate CKD (Jain and Reilly, 2019). Conversely, data for late stages are poor and controversial, in which the risk of bleeding increases with CKD severity (Elenjickal, 2024). But warfarin remains the primary treatment for end-stage renal disease, with anticoagulant therapy decisions being highly individualized. Currently, DOACs are metabolized by the kidney with varying degrees, which can lead to drug accumulation and increased bleeding risk in patients with renal insufficiency. Dabigatran etexilate is highly dependent on renal excretion, and about 80% of plasma dabigatran etexilate is excreted through the kidney in its original form. Compared with other available direct oral anticoagulants, apixaban is minimally dependent on renal clearance, with approximately 27% of the drug being cleared by this route and the remainder by other means (Hindley, 2023). Different DOACs have varying metabolic pathways and renal clearance. At the same time, the PK/PD of DOACs is also different with different eGFR levels. Many phase III clinical trials of DOACs exclude patients with CKD stage 4, making the evidence for DOACs in patients with CKD even more scarce (Aursulesei and Costache, 2019). Therefore, we assessed the clinical efficacy and safety of warfarin versus DOACs through published randomized controlled trials (RCT) and conducted a meta-analysis.

## 2 Materials and methods

We conducted a meta-analysis of RCTs in accordance with the guidelines outlined in the Preferred Reporting Items for Systematic Reviews and Meta-Analyses (PRISMA).

### 2.1 Search strategy

An exhaustive literature search was independently performed by two researchers utilizing PubMed, Embase, Web of Science, the Cochrane Library, and the ClinicalTrials.gov database, encompassing studies from the inception of each up to 30 June 2024. The computer-based searches employed a combination of keywords and keyword phrases pertinent to the study participants (such as CKD, AF, VTE, CKD with VTE or AF), drugs (such as warfarin, dabigatran, rivaroxaban, apixaban, or edoxaban), and results (such as VTE recurrence, stroke, intracranial bleeding, or gastrointestinal bleeding). The references lists of all retrieved articles were manually examined to identify additional potentially relevant research. This study is registered with PROSPERO (CRD42024510727).

### 2.2 Inclusion and exclusion criteria

The inclusion criteria were as follows: RCTs, Adult with CKD (Creatinine clearance <60 mL/min or eGFR <60 mL/min/1.73 m^2^), evaluated the clinical efficacy and safety of warfarin versus DOACs in CKD with VTE or AF; Patients had to be randomly treated with warfarin or DOACs to be included. The outcomes of this systematic review are thrombosis recurrence or VTE-related deaths and major bleeding in CKD patients with VTE, stroke or systemic embolism and major bleeding in CKD patients with AF. Life-threatening bleeding was defined as fatal bleeding. There was no limit to the length of follow-up for included studies. Exclusion criteria include: duplicate articles, inconsistent research content, review articles, meeting abstracts, animal studies, case reports, research programs, non-English or non-Chinese articles, non-randomized and observational studies, and RCTs with placebo controls.

### 2.3 Data extraction and risk of bias assessment

Two reviewers (Y.Q.N and Z.X.Y) independently screened the titles and abstracts, subsequently conducting full-text reviews to identify articles that met inclusion criteria. Following this, study characteristics, baseline characteristics, and prespecified efficacy and safety outcomes were extracted from the selected studies. The included studies were then evaluated using The Cochrane Risk of Bias criteria (Higgins, 2011).

### 2.4 Statistical methods

We computed the relative risk (RR) and 95% confidence interval (CI) for each study. Heterogeneity was evaluated using the I^2^ statistic. In cases where I^2^ ≤ 50% and p ≥ 0.1, indicating no significant heterogeneity among the findings, a fixed-effect model was employed. Conversely, when I^2^ > 50% and p < 0.1, suggesting significant heterogeneity among the results, a random-effects model was utilized (Higgins, 2003). If the event rate is zero, a half-integer continuity correction was applied to all four cells (Friedrich et al., 2007). Publication bias was evaluated using visual funnel plots and Egger test, with the trim-and-fill method applied if any publication bias was detected (Duval and Tweedie, 2000). Sensitivity analysis was conducted by excluding one study at a time to ensure result stability. All statistical analyses were performed using Revman software (version 5.3). *p* < 0.05 was considered statistically significant.

### 2.5 Subgroup analysis

To evaluate the safety and efficacy of anticoagulants in patients with severe CKD, we extracted and analyzed outcome data specifically from this patient subgroup. Severe renal insufficiency was defined as creatinine clearance <30 mL/min or eGFR <30 mL/min/1.73 m^2^, or dialysis-dependent ESKD.

## 3 Result

### 3.1 Study identification and characteristics

**FIGURE 1:**
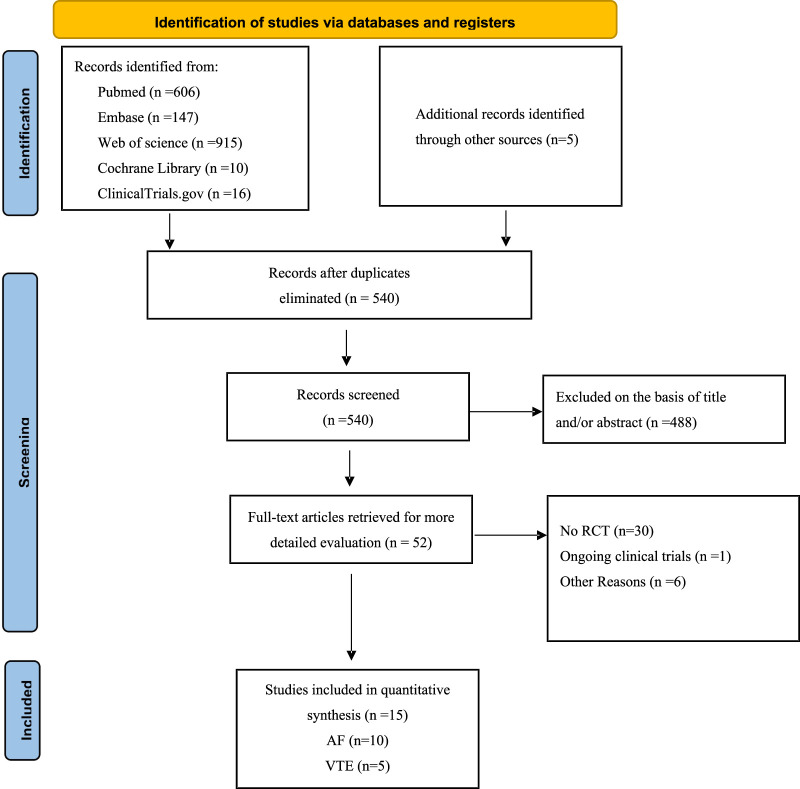
Flow chart of the selected study.

By June 2024, 540 citations were identified (Figure 1). After excluding 488 irrelevant studies based on title/abstract, 30 non-RCT studies, one ongoing clinical trial, and six articles for other reasons, 15 RCTs were included. A PRISMA-based flowchart (Figure 1) and checklist (Supplementary Table S1) were provided. Study characteristics are detailed in Table 1. Among the 15 included studies, the intervention group encompassed various DOACs, comprising rivaroxaban (in five trials), dabigatran etexilate (in three trials), edoxaban (in two trials), and apixaban (in five trials), all studies used warfarin as a comparator. The 15 randomized controlled trials involved 16,361 patients. Of these, 10 trials included 14,491 participants with AF, with a median sample size of 230 participants (range 90–4,074 participants) and a median follow-up duration of 20.25 months (range 1–33.6 months). Five trials involved 1,870 participants with acute VTE, with a median sample size of 327 participants (range: 108–657 participants) and a median follow-up duration of 12 months (range: 6–36 months).

**TABLE 1:** Characteristics of the included studies.

Study name, author, year	Inclusion criteria	Participants, n		Intervention	GFR estimation equation	Renal function (eGFR/CrCl)	Follow-up, months	Primary efficacy outcome	Primary safety outcome
		Warfarin	DOACs						
Trials involving participants with AF									
ARISTOTLE, Granger (2011)	AF or flutter with at least one risk factor for stroke	1,515	1,502	Apixaban 5 mg bid	NS	CrCl<50 mL/min	22.5 (median)	Stroke or systemic embolism	Major bleeding
RENAL-AF, Pokorney (2022)	AF and CHA2DS2-VASc scores ≥2	72	82	Apixaban 5 mg bid or 2.5 mg bid (If age ≥80 years and/or weight ≤60 kg)	Cockroft-Gault	Chronic hemodialysis	11 (apixaban group) and 11.3 (warfarin group)	Stroke and systemic embolism	Major or CRNM bleeding
AF-TIMI 48 Trial, Bohula (2016)	AF and CHADS2 risk score ≥2	1,361	2,713	Edoxaban 60 mg qd or 30 mg qd, or 15 mg qd, (If CrCl 30–50 mL/min, ≤60 kg, or potent P-gp inhibitor used during screening/trial)	Cockroft-Gault	CrCl 30–50 mL/min	33.6 (median)	Stroke or systemic embolism	Time to the first occurrence of major bleeding
De Vriese (2021)	NVAF and CHADS2 risk score ≥2	44	46	Rivaroxaban 10 mg qd	NS	Chronic hemodialysis	22.5 (median)	Composite of fatal cardiovascular disease and nonfatal stroke, cardiac events, and other vascular events	Bleeding events
ROCKET AF, Patel (2011)	NVAF and moderate to high risk of stroke	1,456	1,485	Rivaroxaban 15 mg qd	Cockcroft-Gault	CrCl 30–49 mL/min	23.5 (median)	Stroke or systemic embolism	Major or CRNM bleeding
J-ROCKET AF Hori (2013)	NVAF and moderate to high risk of stroke, prior history of stroke or CHADS2 score ≥2	143	141	Rivaroxaban 10 mg qd	Cockcroft-Gault	CrCl 30–49 mL/min	1	Stroke and non-CNS systemic embolism	Major or CRNM bleeding
RE-LY Hijazi (2014)	AF with at least one risk factor for stroke	1,126	2,428	Dabigatran etexilate 110 or 150 mg bid	Cockcroft-Gault, CKD-EPI and cystatin C	CrCl <50 mL/min	24 (median)	Stroke or systemic embolism	Major bleeding
Chashkina (2020)	NVAF	36	73	Rivaroxaban 15 mg qd	CKDEPI	eGFR 15–29 mL/min/1.73 m^2^	18 (mean)	Ischemic stroke and systemic embolism	Major, minor, and clinically relevant minor bleeding
Arun Kumar et al. (2024)	AF or atrial flutter	46	46	Apixaban 5 mg bid	—	eGFR <60 mL/min/1.73 m^2^	12	Ischemic stroke incidences, recurrent thromboembolism incidences and TTR	Major bleeding
Gita Bipin Chandra and Singh (2023)	AF or atrial flutter	88	88	Apixaban 5 mg bid	—	eGFR <60 mL/min/1.73 m^2^	12	Ischemic stroke incidences, recurrent thromboembolism incidences and TTR	Major bleeding
Trials involving participants with VTE									
RE-COVER I/Ⅱ, Goldhaber (2017)	Symptomatic, proximal DVT of the legs or PE	123	114	5 days of parenteral anticoagulant followed by dabigatran etexilate 150 mg bid	Cockcroft-Gault	CrCl 30–49 mL/min	6	Symptomatic, recurrent VTE/VTE-related death	Major bleeding
EINSTEIN DVT and EINSTEIN EINSTEIN Investigators (2010)	Symptomatic DVT and/or PE	324	333	Rivaroxaban 15 mg bid for 3 weeks, followed by 20 mg	NS	CrCl <50 mL/min	12	Symptomatic, recurrent VTE (composite of DVT or nonfatal or fatal PE)	Major bleeding
Hokusai-VTE, 2013 (Hokusai, 2013)	Symptom DVT and symptomatic PE (with or without DVT)	273	268	5 days of parenteral anticoagulant followed by Edoxaban 30 mg qd	NS	CrCl 30–50 mL/min	12	Symptomatic, recurrent VTE (composite of DVT or nonfatal or fatal PE)	Major or CRNM bleeding
RE-MEDY, Schulman (2013)	Symptomatic, proximal DVT or PE	49	59	Dabigatran etexilate 150 mg bid	Cockcroft-Gault	CrCl <50 mL/min	36	Symptomatic, recurrent VTE/VTE-related death	Major or CRNM bleeding
AMPLIFY, Agnelli (2013)	symptomatic proximal DVT or PE (with or without DVT)	158	169	Apixaban, 10 mg bid for 7 days followed by 5 mg bid	NS	CrCl <50 mL/min	6	Symptomatic, recurrent VTE/VTE-related death	Major bleeding

DOACs, direct oral anticoagulants; GFR, glomerular filtration rate; CrCl, creatinine clearance; NS, not stated; AF, atrial fibrillation; CRNM, bleeding, clinically relevant nonmajor bleeding; P-gp, phosphorylated-glycoprotein; NVAF, non-valvularatrial fibrillation; VTE, venous thromboembolism; DVT, deep venous thrombosis; PE, pulmonary embolism; non-CNS, non-central nervous system; eGFR, estimated glomerular filtration rate.

### 3.2 Patient characteristics

Data were reported for 3,922 participants out of a total of 14,491 participants with renal insufficiency comorbid with AF. The mean age ranged from 61.6 to 78 years in the DOACs group and from 59.8 to 78 years in the warfarin group (Table 2). Mean CHADS2 scores ranged from 3.56 to 4.77 in the DOACs group and from 3.52 to 4.83 in the warfarin group. Additionally, the proportion of patients with a history of stroke varied significantly, with the DOACs group exhibiting a range of 10.0%–65.9% and the warfarin group showing a range of 16.7%–57.3%. However, since the data pertaining to participants with renal insufficiency and VTE in those studies were extracted from subgroup analyses, it was noted that information on the characteristics of these patients was unavailable.

**TABLE 2:** Patient characteristics in included studies for AF.

Study name, author, year	Mean age (years)		Female (%)		Hypertension (%)		Diabetes (%)		Mean CHA2DS2-VASc score		Stroke history (%)		Gastrointestinal bleeding history (%)	
	Warfarin	DOACs	Warfarin	DOACs	Warfarin	DOACs	Warfarin	DOACs	Warfarin	DOACs	Warfarin	DOACs	Warfarin	DOACs
ARISTOTLE, Granger (2011)	70	70	35	35.5	—	—	—	—	—	—	—	—	—	—
RENAL-AF, Pokorney (2022)	68	69	30.6	41.5	93.1	96.3	65.3	51.2	4	4	16.7	20.7	NS	NS
AF-TIMI 48 Trial, Bohula (2016)	—	—	—	—	—	—	—	—	—	—	—	—	—	—
De Vriese (2021)	80.3 (median)	79.9 (median)	43.2	23.9	—	—	45.5	43.5	4.8	4.7	36.4	36.2	27.3	19.6
ROCKET AF, Patel (2011)	—	—	—	—	—	—	—	—	—	—	—	—	—	—
J-ROCKET AF, Hori (2013)	78	78	33.6	25.5	82.5	82.3	25.9	36.2	3.52	3.56	57.3	65.9	—	NS
RE-LY, Hijazi (2014)	—	—	—	—	—	—	—	—	—	—	—	—	—	—
Chashkina (2020)	78	77	61	56	96	98	44	37	4.7	4.6	36.0	10.0	—	—
Arun Kumar et al. (2024)	59.8	61.6	47.8	45.6	—	—	—	—	4.83	4.65	—	—	—	—
Gita Bipin Chandra and Singh (2023)	61.7	63.5	51.1	47.7	—	—	—	—	4.71	4.77	—	—	—	—

DOACs, direct oral anticoagulants.

### 3.3 Risk of bias

Of the ten trials reporting stroke or systemic embolism in patients with AF, the method employed for randomized sequence generation was deemed high-risk or unclear in five of the studies. In terms of allocation concealment methods, eight studies used low-risk methods, whereas the methods used in the remaining two studies were not clearly specified. Five studies were at high risk for bias due to being open-label trials, lacking blinding of participants and personnel.

Trials involving participants (Pokorney, 2022; De Vriese, 2021; Chashkina, 2020; Stanifer, 2020) with AF on hemodialysis or with severe renal insufficiency, generally had high or unclear bias risks in random sequence generation and participant blinding.

All trials reporting VTE or VTE-related deaths in VTE patients used a low-risk method for randomized sequence generation and allocation concealment. One trials (Investigators, 2010) was high-risk for blinding as it was open-label, whereas the other four studies were low-risk. Summary of risk of bias assessments are shown in Supplementary Figures S1–S3. A potential publication bias was found in trials of AF patients with CKD reporting non-hemorrhagic strokes (P_Egger_ = 0.01), but not in other meta-analyses. Egger’s test results are in Supplementary Tables S2–S4.

### 3.4 Sensitivity analyses

When heterogeneity is high (I^2^ ≥ 50%) in the meta-analysis, we perform sensitivity analysis to check result robustness. The analysis showed no single trial significantly affected the overall results (Supplementary Tables S5–S7).

### 3.5 Effective outcome in atrial fibrillation

#### 3.5.1 Stroke or systemic embolism

**FIGURE 2:**
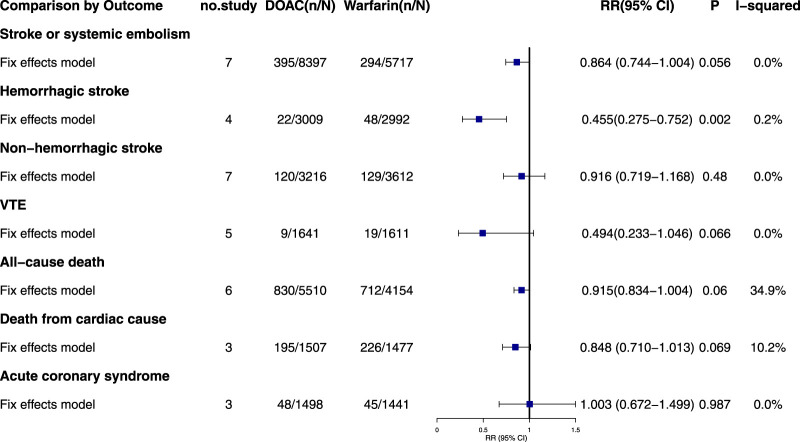
Summary of effective outcomes in patients with CKD combined with AF.

Seven studies reported stroke or systemic embolism events with no significant heterogeneity (I^2^ = 0.0%, P = 0.429). The fixed-effects model showed non-significant results (RR = 0.864, 95% CI = 0.744–1.004, P = 0.056) (Figure 2; Supplementary Figure S4). Although the Egger test was not significant (P_Egger_ = 0.288), the asymmetrical visual funnel plot indicated potential publication bias. Including four virtual trials with the trim-and-fill method increased the RR by 0.176 (RR = 1.040, 95% CI = 0.842–1.284, P = 0.719), confirming the robustness of the analysis.

#### 3.5.2 Hemorrhagic stroke

Four studies on hemorrhagic stroke showed consistent results (I^2^ = 0.2%, P = 0.391). A fixed-effects model indicated a significant reduction in risk with DOACs compared to warfarin (RR = 0.455, 95% CI: 0.275–0.752, P = 0.002). The symmetrical funnel plot and Egger test (P_Egger_ = 0.915) suggest no publication bias.

#### 3.5.3 Non-hemorrhagic stroke

Seven studies reported non-hemorrhagic stroke events with no significant heterogeneity (I^2^ = 0.0%, P = 0.528). The fixed-effects model showed no statistically significant results (RR = 0.916, 95% CI: 0.719–1.168, P = 0.480). Despite Egger’s test indicating publication bias (P_Egger_ = 0.001), the “Trim-and-Fill” method confirmed the pooled outcomes remained stable (RR = 0.962, 95% CI = 0.757–1.222, P = 0.750).

#### 3.5.4 VTE

Five studies documented venous thrombosis events in patients with AF, including two double-zero studies (Pokorney, 2022; De Vriese, 2021). Corrections were made using half-integer continuity, and five studies were finally included. The combined heterogeneity was minimal (I^2^ = 0.0%, P = 0.983), and the results were not statistically significant (RR = 0.494, 95% CI: 0.233–1.046, P = 0.066). Egger’s test showed no significant publication bias (P_Egger_ = 0.552).

#### 3.5.5 All-cause death

Six studies on all-cause mortality in AF patients showed no significant pooled heterogeneity (I^2^ = 34.9%, P = 0.175). The fixed-effects model indicated non-significant results (RR = 0.915, 95% CI: 0.834–1.004, P = 0.060). Both the Egger test and funnel plot revealed no significant publication bias (P_Egger_ = 0.509).

#### 3.5.6 Death from cardiac cause

Only three studies reported cardiac-related deaths, showing low combined heterogeneity (I^2^ = 10.2%, P = 0.328). The fixed-effects model indicated no statistically significant results (RR = 0.848, 95% CI: 0.710–1.013, P = 0.069) and no significant differences. The visual funnel plot showed no obvious asymmetry (P_Egger_ = 0.566).

#### 3.5.7 Acute coronary syndrome

Three studies on acute coronary syndrome showed no significant heterogeneity (I^2^ = 0.0%, P = 0.461). Using a fixed-effects model, the results were not statistically significant (RR = 1.003, 95% CI: 0.672–1.499, P = 0.987). The Egger test showed no significant publication bias (P_Egger_ = 0.155), though the funnel plot indicated slight asymmetry. The results remained consistent after trim-and-fill adjustment (RR = 1.001, 95% CI: 0.669–1.499, P = 0.994).

### 3.6 Safety outcome in atrial fibrillation

#### 3.6.1 Major bleeding

**FIGURE 3:**
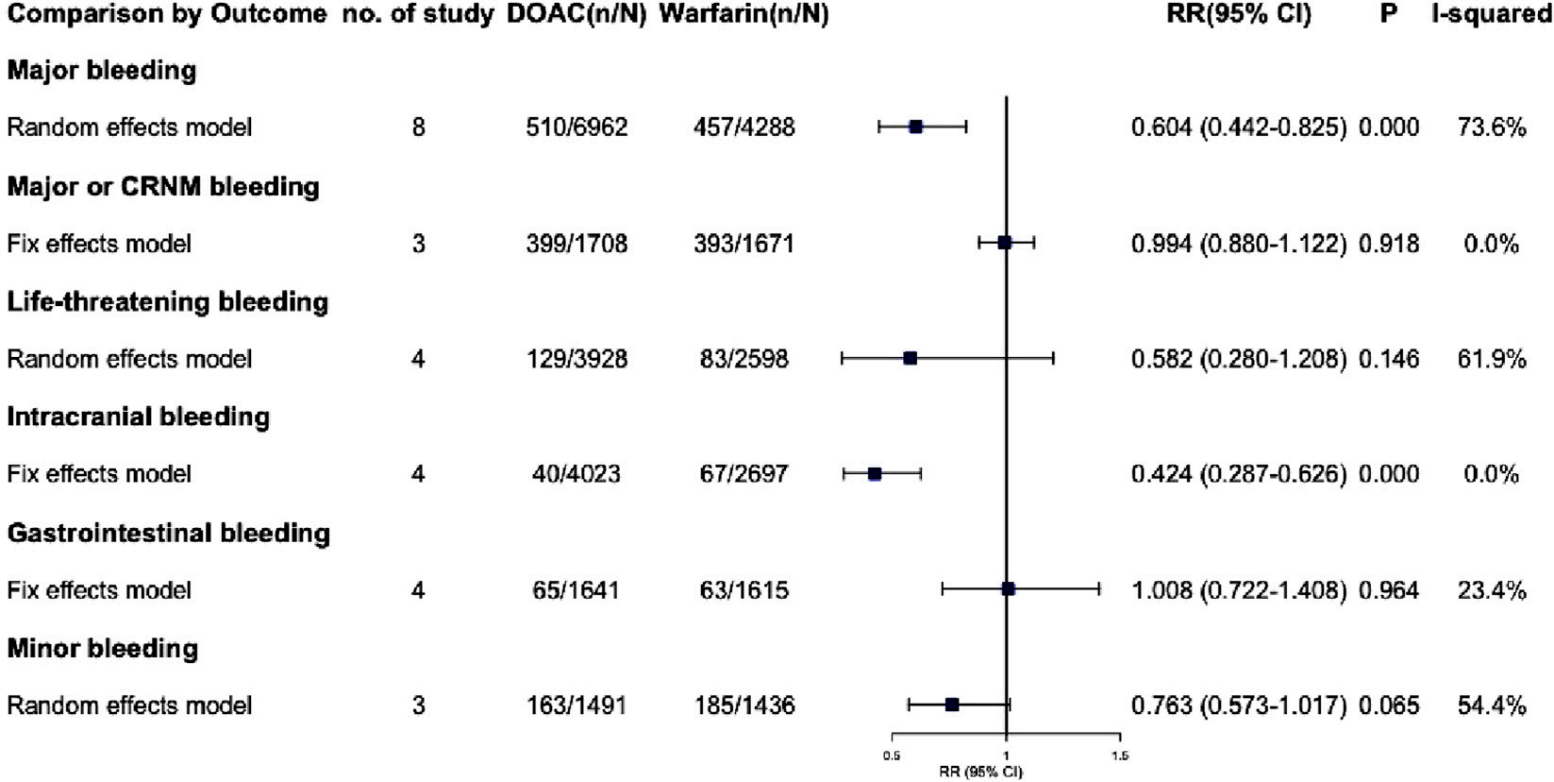
Summary of safety outcomes in patients with CKD combined with AF.

Eight trials reported major bleeding events, showing high heterogeneity (I^2^ = 73.6%, P = 0.000). Using a Random-effects model, the results were statistically significant (RR = 0.604, 95% CI: 0.442–0.825, P = 0.002) (Figure 3; Supplementary Figure S5), indicating that AF patients with renal dysfunction had a lower risk of major bleeding with DOAC compared to warfarin. Due to the high heterogeneity, a sensitivity analysis was performed using the one-by-one exclusion method. The findings of this analysis, as detailed in Supplementary Table S5, confirms the robustness of the findings as excluding any single study did not significantly change the overall results. The Egger test showed no significant publication bias (P_Egger_ = 0.388), though the funnel plot appeared slightly asymmetrical. After adjusting with the trim and fill method, two virtual trials were included (RR = 0.668, 95% CI: 0.493–0.906, P = 0.009).

#### 3.6.2 Major or CRNM bleeding

Three studies examined the incidence of major or CRNM bleeding in patients with AF and CKD. The pooled analysis showed that DOACs and warfarin had similar risks for major or CRNM bleeding, with no significant difference (RR = 0.994, 95% CI: 0.880–1.122, P = 0.918) and no significant heterogeneity (I^2^ = 0.0%, P = 0.461). The funnel plot and Egger’s test indicated no publication bias (P_Egger_ = 0.265).

#### 3.6.3 Life-threatening bleeding

Four studies reported life-threatening bleeding events with high heterogeneity (I^2^ = 61.9%, P = 0.049). Using a random-effects model, the results were not statistically significant (RR = 0.582, 95% CI: 0.280–1.208, P = 0.146). Sensitivity analysis (Supplementary Table S5) showed that excluding the RE-LY trial (Hijazi, 2014) reduced heterogeneity to 0.0%. A fixed-effects model then showed statistically significant results (RR = 0.39, 95% CI: 0.21–0.74, P = 0.004). The Egger test indicated no significant publication bias (P_Egger_ = 0.781).

#### 3.6.4 Intracranial bleeding

Four studies on intracranial bleeding showed no significant heterogeneity (I^2^ = 0.0%, P = 0.859) and were pooled using a fixed-effect model (RR = 0.424, 95% CI: 0.287–0.626, P = 0.000), indicating a significantly reduced risk with DOAC. No publication bias was detected (P_Egger_ = 0.360).

#### 3.6.5 Gastrointestinal bleeding

Four studies on gastrointestinal bleeding were pooled (I^2^ = 23.4%, P = 0.271) and analyzed using a fixed-effect model (RR = 1.008, 95% CI: 0.722–1.408, P = 0.964). The findings showed no significant difference in the risk of gastrointestinal bleeding between DOACs and warfarin.

#### 3.6.6 Minor bleeding

Minor bleeding incidents were reported in three studies with significant heterogeneity (I^2^ = 54.4%, P = 0.112). Random effects models showed non-significant results (RR = 0.763, 95% CI: 0.573–1.017, P = 0.065). A sensitivity analysis was performed due to the high heterogeneity, and detailed results are in Supplementary Table S5. Excluding the study of De Vriese (2021) changed the RR from 0.763 (95% CI: 0.573–1.017) to 0.758 (95% CI: 0.622–0.924, P = 0.006), but high heterogeneity persisted (I^2^ = 49.6%, P = 0.159). Three trials were included, and results should be interpreted cautiously. No publication bias was detected (P = 0.666).

### 3.7 Effective outcome in VTE

#### 3.7.1 Recurrent VTE or VTE-related deaths

**FIGURE 4:**
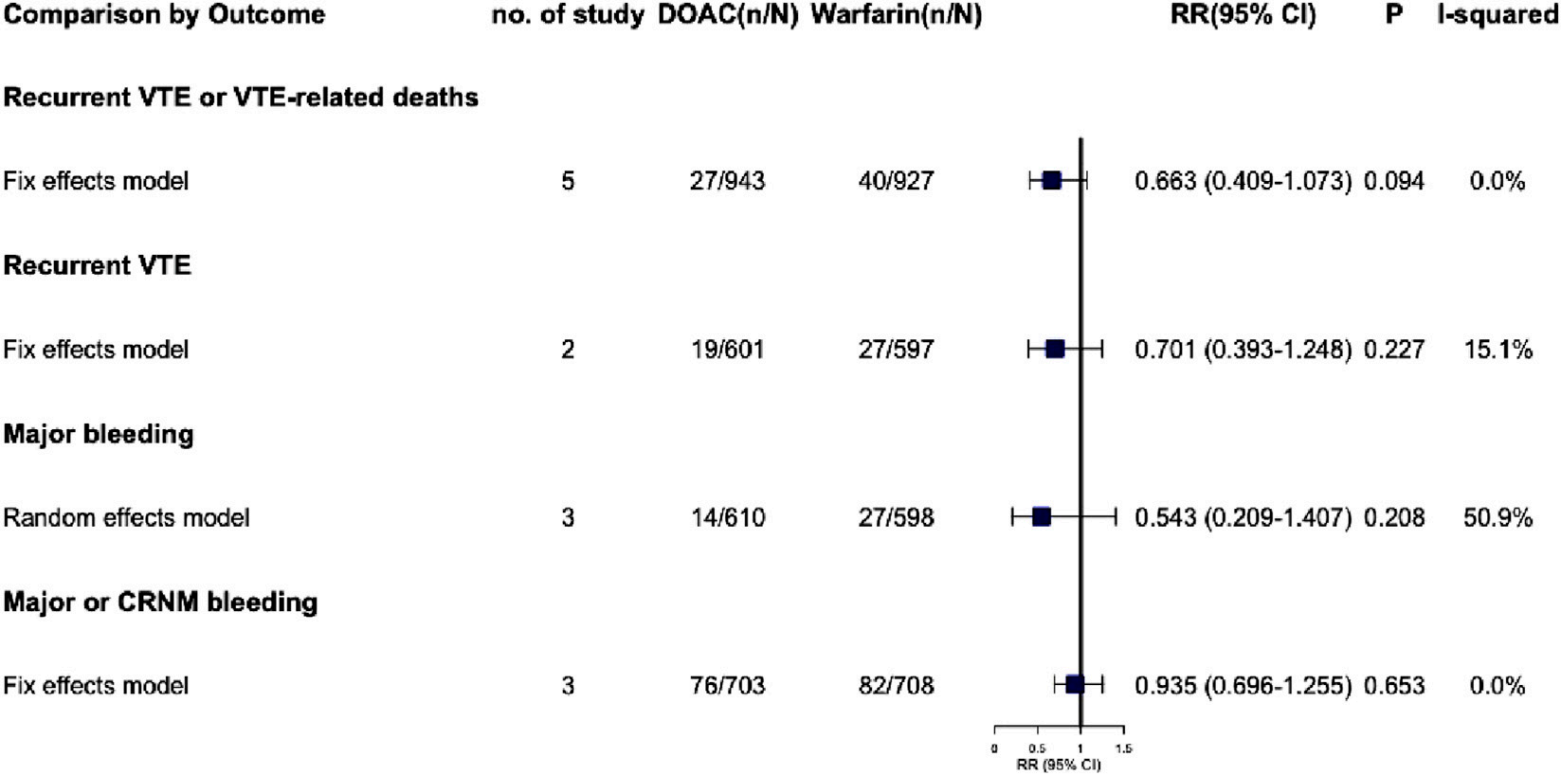
Summary of effective and safety outcomes in patients with CKD combined with VTE.

All five studies reported recurrent VTE or VTE-related deaths, with no significant difference between the warfarin and DOAC groups (RR = 0.663, 95% CI: 0.409–1.073, P = 0.094, I^2^ = 0.0%) (Figure 4; Supplementary Figure S6). Visual funnel plots showed no symmetry (P_Egger_ = 0.435).

#### 3.7.2 Recurrent VTE

Two studies were analyzed, showing no statistically significant differences (RR = 0.701, 95% CI: 0.393–1.248, P = 0.227, I^2^ = 15.1%), and no publication bias was found.

### 3.8 Safety outcomes in VTE

#### 3.8.1 Major bleeding

Three studies reported significant bleeding events in VTE patients with renal dysfunction, showing high heterogeneity (I^2^ = 50.9%, P = 0.130). A random-effects model found no significant difference (RR = 0.543, 95% CI: 0.209–1.407, P = 0.208), indicating similar major bleeding risks for DOACs and warfarin in these patients. Due to the high heterogeneity, each study was excluded one by one to assess their impact on the meta-results. Sensitivity analysis revealed that excluding RE-COVER I/II (Goldhaber, 2017), reduced heterogeneity to 0.0% and significantly altered the meta-analysis results, with RR dropping from 0.543 to 0.363 (95% CI: 0.161–0.819, P = 0.015, see Supplementary Table S6). However, the small sample size warrants cautious interpretation of these findings.

#### 3.8.2 Major or CRNM bleeding

Three studies included in the meta-analysis indicated that patients with renal dysfunction induced by DOAC or warfarin had a comparable risk of major or CRNM bleeding events (RR = 0.935, 95% CI: 0.696–1.255, P = 0.653, I^2^ = 0.0%). The visual funnel plot showed no significant asymmetry (P_Egger_ = 0.816).

### 3.9 Subgroup

**FIGURE 5:**
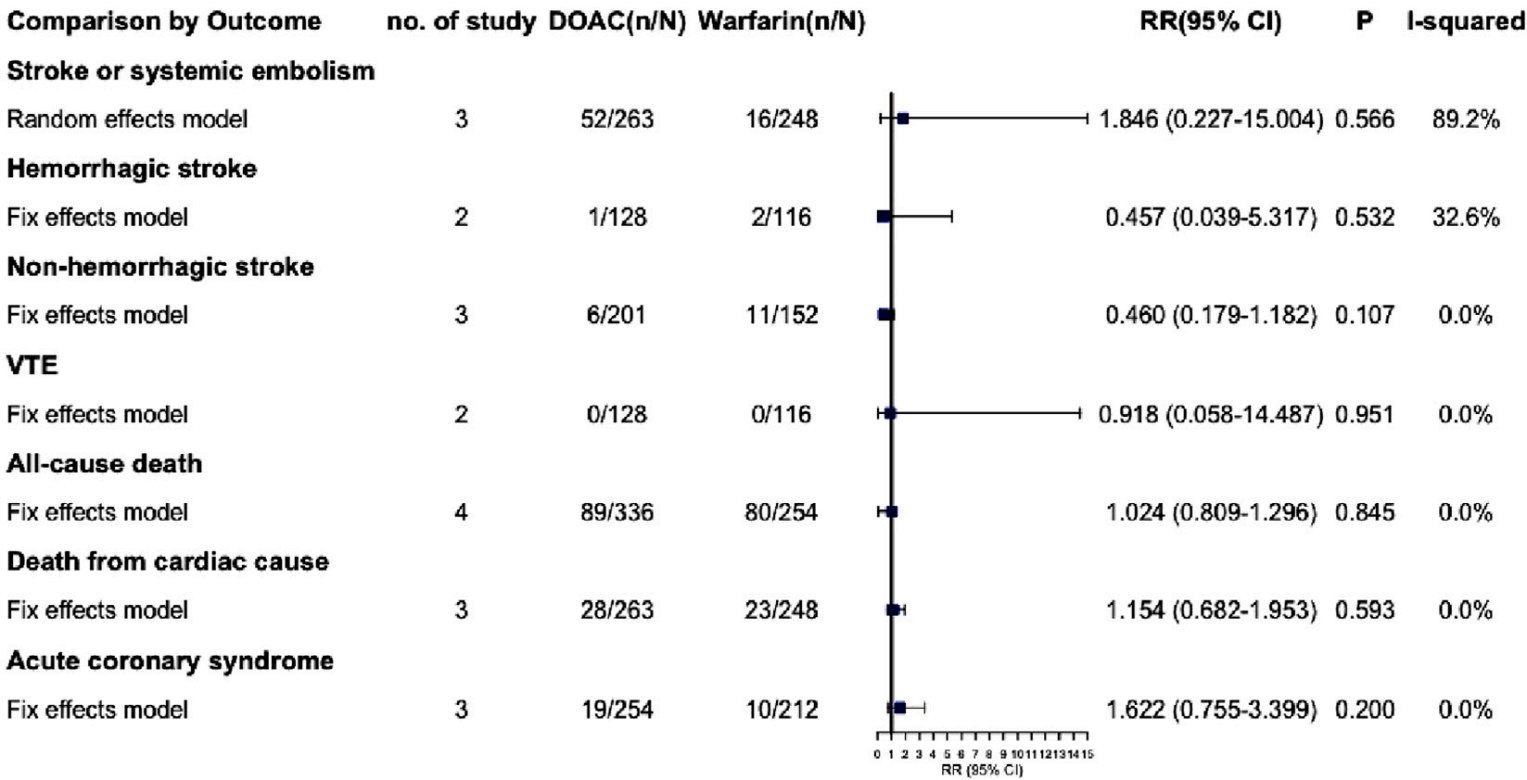
Summary of effective outcomes in patients with severe CKD combined with AF.

**FIGURE 6:**
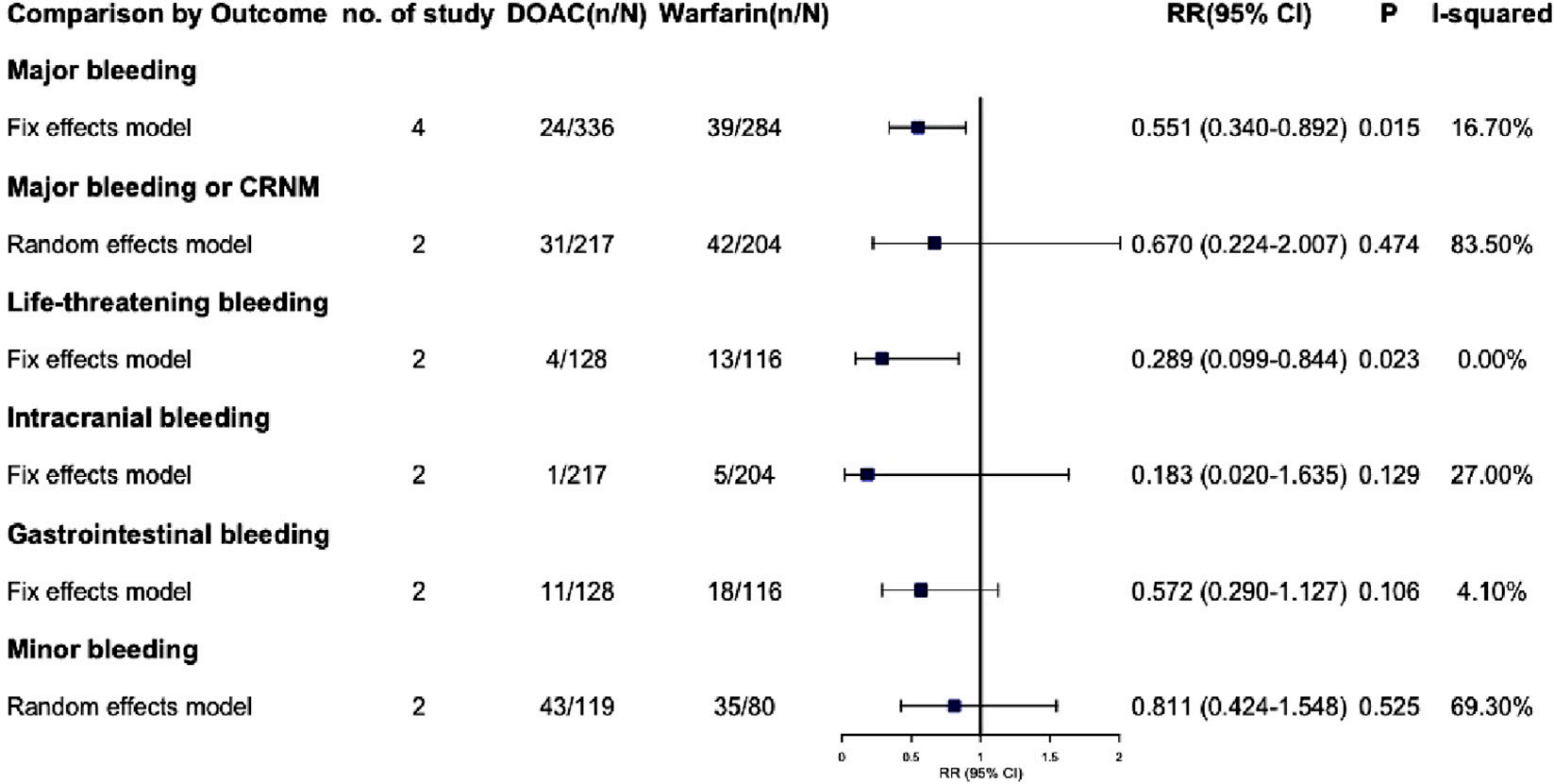
Summary of safety outcomes in patients with severe CKD combined with AF.

Due to the lack of available data, we were unable to extract outcome data specifically for VTE complicated with severe renal insufficiency. Consequently, we have only extracted outcome data for AF complicated with severe renal insufficiency. The effective outcomes include stroke or systemic embolism, hemorrhagic stroke, non-hemorrhagic stroke, VTE, all-cause death, death from cardiac cause and acute coronary syndrome, Among these outcomes, DOACs did not demonstrate statistically significant differences compared to warfarin (P > 0.05) (Figure 5; Supplementary Figure S8). Safety outcomes include various types of bleeding: major, CRNM, life-threatening, intracranial, gastrointestinal, and minor. DOACs showed statistically significant differences from warfarin in major bleeding and life-threatening bleeding (RR: 0.551 [95% CI: 0.340–0.892], P = 0.015; RR: 0.289 [95% CI: 0.099–0.844], P = 0.023, respectively) (Figure 6; Supplementary Figure S9). Other bleeding outcomes did not show significant differences. Detailed data are available in the Supplementary Material.

## 4 Discussion

AF, VTE, and CKD frequently coexist, presenting a common challenge in clinical practice. The determination of suitable anticoagulant therapy in these instances presents considerable complexity. Oral anticoagulation is an efficacious strategy for reducing the incidence of ischemic stroke and systemic embolism in AF patients, as well as for mitigating the morbidity and mortality associated with VTE. However, despite the well-documented benefits for the majority of patients, these advantages have not been as clearly demonstrated in individuals with CKD due to the delicate balance between the bleeding risks and thromboembolic complications (Jones, 2024). DOACs are increasingly preferred over VKAs for the management of patients with AF and compromised renal function, given that VKAs have been linked to the accelerated progression of chronic kidney disease. However, DOACs are subject to renal elimination to varying extents, necessitating dosage adjustments based on the patient’s renal function. Furthermore, it has been proposed that VKAs may exacerbate the progression of CKD due to their role in promoting atherosclerotic plaque formation and vascular and soft tissue calcification through their pharmacological mechanisms (Hahn and Lamparter, 2023). CKD has been identified as an independent risk factor for VTE. The standard treatment regimen for VTE in patients with CKD predominantly involves initial anticoagulant therapy with unfractionated heparin (UFH) or low molecular weight heparin (LMWH), serving as a bridge to oral VKA therapy for a duration of 3 months or longer. DOACs have been demonstrated to be effective in both the initial and extended treatment phases for patients with VTE and mild to moderate CKD (CrCl 30–80 mL/min) (Dobrowolski et al., 2017; Kakkos, 2021). Overall, various direct oral anticoagulants DOACs exhibit distinct metabolic pathways and renal clearance mechanisms. Concurrently, the pharmacokinetics (PK) and pharmacodynamics (PD) of DOACs vary with different estimated glomerular filtration rate (eGFR) levels. Consequently, for patients with AF or VTE who also have CKD, the selection of DOACs and the determination of appropriate dosages should be individualized. In contrast, the dosing of warfarin is determined and adjusted based on the international normalized ratio (INR). The 2021 Clinical Practice Guidelines on the Management of Venous Thrombosis by the European Society for Vascular Surgery (ESVS) primarily underscore the importance of routine assessment of patients’ renal function but fall short of offering detailed recommendations (Kakkos, 2021). In contrast, 2021 European Heart Rhythm Association Practical Guide on the Use of Non-Vitamin K Antagonist Oral Anticoagulants in Patients with Atrial Fibrillation provide comparatively more specific directives for each DOAC (Steffel, 2021).

Among the 15 studies included in this review, only 5 RCTs investigated patients with VTE combined with CKD. A substantial portion of the studies concentrated on patients with AF, evaluating the efficacy and safety of warfarin and DOACs in individuals with renal insufficiency. In AF patients with CKD, seven studies reported stroke or systemic embolism events, showed non-significant results (RR = 0.864, P = 0.056). In a recent meta-analysi^s^ (Li, 2024) incorporating 9 RCTs, it was demonstrated that DOACs could reduce the risk of stroke or systemic embolism in CKD patients with AF (RR = 0.75, P < 0.001). This finding diverges from our study results, which may stem from two factors: first, their analysis included a trial involving phenprocoumon (Reinecke, 2023) that we excluded; second, our study incorporated the latest three RCTs, thereby contributing to the discrepancy in outcomes. Four studies reported hemorrhagic stroke events, DOACs were found to be associated with a lower risk of hemorrhagic stroke than warfarin (RR = 0.455, 95% CI: 0.275–0.752, P = 0.002). No significant difference was observed between warfarin and DOACs in the incidence of ischemic stroke. Eight trials reported major bleeding events, and the use of DOACs was associated with a lower risk of major bleeding compared to warfarin among patients with AF and CKD (RR = 0.604, 95% CI: 0.442–0.825, P = 0.002). This finding is consistent with another meta-analysis that included RCTs and cohort studies conducted over the past 22 years (Parker, 2022). In addition, in life-threatening bleeding, the data were statistically significant after deletion of the RE-LY study due to high heterogeneity (RR = 0.396, 95% CI: 0.210–0.749, P = 0.004). The analysis may be due to the fact that a total of 6,526 patients are included in the results of life-threatening bleeding, and more than half of the patients involved in this study alone (3553/6526, 54.4%), which may greatly affect the results of RE-LY study. Additionally, the FDA recommends a reduced dose of dabigatran for patients with a CrCl of 30–50 mL/min, with a recommended dose of 75 mg twice daily. However, the doses utilized in this trial were 110 mg or 150 mg twice daily, which may have introduced potential bias (Hariharan and Madabushi, 2012). Four studies demonstrated that DOACs significantly reduced the risk of intracranial bleeding (RR = 0.424, 95% CI: 0.287–0.626, P < 0.001). Additionally, a national cohort study evaluating the safety and efficacy of warfarin or rivaroxaban compared to apixaban in patients with advanced CKD and atrial fibrillation yielded similar findings. Specifically, apixaban was associated with a lower risk of intracranial bleeding compared to rivaroxaban (Fu, 2024).

We performed a subgroup analysis on patients with AF and severe CKD, including those on dialysis. Results revealed no significant efficacy difference between DOACs and warfarin. Regarding safety, DOACs seemed to have a lower risk of major and life-threatening bleeding (RR: 0.551 [95% CI: 0.340–0.892], P = 0.015; RR: 0.289 [95% CI: 0.099–0.844], P = 0.023, respectively). However, due to limited data, fully clarifying DOAC safety in severe CKD patients is challenging. Thus, we are extremely cautious about using DOACs, especially dabigatran (80% renal excretion), in this group.

In the evaluation of VTE with CKD, oral anticoagulants demonstrated no statistically significant impact on the recurrence of thrombosis or the incidence of bleeding. Consistent with a 2020 meta-analysis, DOACs was found to be similar in efficacy and safety to warfarin in patients with stage 4–5 CKD or dialysis (Chen, 2021). Although no significant difference in gastrointestinal bleeding was observed, the risk associated with various anticoagulants varied. The risk of gastrointestinal bleeding varied among anticoagulants: dabigatran and rivaroxaban posed a higher risk than warfarin, while apixaban posed a lower risk (Guo, 2019; Abraham, 2017). The incidence of gastrointestinal bleeding exhibited a dose-dependent relationship, wherein edoxaban administered at 60 mg per day resulted in a higher incidence of gastrointestinal bleeding compared to warfarin, while edoxaban administered at 30 mg per day was associated with a lower incidence of gastrointestinal bleeding relative to warfarin (Aisenberg, 2018). A real-world study of Asian people with atrial fibrillation showed that the risk of hospitalization for gastrointestinal bleeding due to edoxaban was lower than that of warfarin (Lee, 2018).

Although DOACs have demonstrated specific advantages, particularly regarding safety, the role of warfarin among oral anticoagulants remains significant. Warfarin’s metabolism via the liver’s cytochrome P450 (CYP450) enzyme system, independent of renal function, renders it a preferred option for patients with CKD, especially those with ESRD, who require long-term anticoagulation therapy. However, frequent fluctuations in INR necessitate more frequent monitoring of patients to ensure that INR levels remain within a clinically acceptable range (Elenjickal, 2024).

## 5 Strengths and limitations

Numerous studies have investigated the use of anticoagulant drugs in patients with renal insufficiency. This meta-analysis incorporates data from 15 RCTs, encompassing a substantial sample size of 16,361 patients. Notably, five of these trials were published within the past 5 years, providing robust and reliable results. To ensure methodological rigor and mitigate the risk of unreliable outcomes due to varying trial designs, only RCTs were included in this analysis. Additionally, we conducted subgroup analyses focusing on patients with severe renal insufficiency, which yielded clinically meaningful insights. However, it is important to acknowledge certain limitations inherent to this meta-analysis. Large sample studies are concentrated in a few studies, in addition, the literature on VTE is small and the research is early. Additionally, for certain populations such as patients with CKD complicated by VTE and those with severe CKD complicated by AF, the lack of access to individual patient clinical characteristic data precludes the complete elimination of all potential confounding factors within the baseline characteristics. On the other hand, due to limited data included, different DOACs were not compared separately with warfarin. Finally, the incorporation of double-zero studies, coupled with the application of the classical half-integer correction, may have resulted in a marginal overestimation of the event occurrence rates.

## 6 Conclusion

Evidence derived from RCTs indicates that DOACs exhibit superior efficacy and safety compared to warfarin in patients with AF and CKD. Additionally, DOACs exhibit comparable efficacy and safety to warfarin in patients with VTE and CKD. Clinicians and pharmacists should tailor the selection of anticoagulant therapy to the individual patient’s clinical profile.

## Data Availability

The original contributions presented in the study are included in the article/Supplementary Material, further inquiries can be directed to the corresponding authors.
