# Supplementary material for: Efficacy and safety of oral anticoagulants in the treatment of chronic kidney disease with atrial fibrillation or venous thromboembolism: a systematic review and meta-analysis
Source: Front Pharmacol. 2025 Aug 29;16:1615284. doi: 10.3389/fphar.2025.1615284 (PMC12425454; doi:10.3389/fphar.2025.1615284)
Supplement: Supplementary file 1 [file DataSheet1.pdf]

1 Effective outcome in AF with severe CKD

1.1 Stroke or systemic embolism

3 studies investigating the incidence of stroke or systemic embolism in patients with severe renal insufficiency and atrial fibrillation exhibited high post-pooled heterogeneity (I^2 = 89.2%, P = 0.000) and were not statistically significant when analyzed using a random effects model (RR = 1.846, 95% CI: 0.227-15.004, P = 0.566). Sensitivity analysis, which involved excluding the study by Vriese et al.,[1] revealed that the results remained statistically non-significant (RR = 0.637, 95% CI: 0.265-1.531, P = 0.312, I^2 = 0.0%) (See Supplementary Table 7). As detailed in Supplementary Table 4, there was no significant publication bias in the three included studies (P_{Egger} = 0.795).

1.2 Hemorrhagic stroke

2 studies have reported hemorrhagic stroke events in patients with AF and severe renal dysfunction. Pooled results using fixed effects models indicated no significant difference in risk between direct oral anticoagulants (DOACs) and warfarin (RR=0.457, 95% CI: 0.039-5.317, P = 0.532, I^2=32.6%). The visual inspection of the funnel plot suggested approximate symmetry, and the Egger's test could not be performed due to the limited number of included studies.

1.3 Non-hemorrhagic stroke

The pooled results of the 3 studies did not find a difference in the risk of non-bleeding stroke between DOAC and warfarin in AF patients with severe renal insufficiency (RR= 0.460, 95%CI: 0.179-1.182, P = 0.107, I^2=0.0%,).

1.4 VTE

2 studies have reported on VTE events in patients with atrial AF and severe renal insufficiency. Notably, both studies were "double-zero" studies, indicating that neither the DOAC group nor the warfarin group experienced systemic embolic events. A continuous half-correction method was utilized for the analysis, and the difference in outcomes between the groups was not statistically significant (RR = 0.918, 95% CI: 0.058-14.487, P = 0.951, I^2 = 0.0%).

1.5 All-cause death

4 studies were included and the outcome was not statistically significant (RR= 1.024, 95%CI: 0.809-1.296, P = 0.845, I^2=0.0%), additionally, no evidence of potential publication bias was detected (P_{Egger}=0.583).

1.6 Death from cardiac cause

3 studies were incorporated into the analysis, yielding no statistically significant outcomes (RR= 1.154, 95% CI: 0.682-1.953, P = 0.593, I^2 = 0.0%). Additionally, no evidence of potential publication bias was detected (P_{Egger} = 0.886).

1.7 Acute coronary syndrome

3 studies were included, with no statistically significant differences in meta-results (RR=1.622, 95%CI: 0.775-3.399, P = 0.200, I^2=0.0%), and no potential publication bias was found (P_{Egger}=0.139).

2 Safety outcoms in AF with severe CKD

2.1 Major bleeding

Major bleeding events were documented in 4 studies, exhibiting low post-pooled heterogeneity (I^2= 16.7%, P = 0.308) and statistically significant post-pooled differences (RR = 0.551, 95% CI: 0.340-0.892, P = 0.015). These findings indicate that DOACs are less likely to induce major bleeding events in patients with severe renal insufficiency combined with AF.

2.2 Major or CRNM bleeding

2 studies reported major or clinically relevant non-major (CRNM) bleeding events, exhibiting high combined heterogeneity when analyzed using random-effects models (RR = 0.670, 95% CI: 0.224-2.007, P = 0.474, I^2 = 83.5%). Given the limited number of studies included, the robustness of these results could not be confirmed through sensitivity analysis. Consequently, these findings should be interpreted with caution.

2.3 Life-threatening bleeding

2 studies characterized by low post-merger heterogeneity (I^2 = 0.0%, P = 0.702) were included in the analysis. The meta-analysis results indicated that DOACs were associated with a significantly lower risk of life-threatening bleeding in patients with severe renal insufficiency compared to warfarin (RR = 0.289, 95% CI: 0.099-0.844, P = 0.023).

2.4 Intracranial bleeding

2 studies were included in the analysis, and the combined results were not statistically significant (RR= 0.183, 95%CI: 0.020-1.635, P = 0.129, I^2= 27.0%).

2.5 Gastrointestinal bleeding

2 studies were included in the analysis, and the combined results were not statistically significant (RR=0.572, 95%CI： 0.290-1.127, P =0.106, I^2= 4.1%).

2.6 Minor bleeding

2 studies were included in the analysis, and the combined results were not statistically significant (RR=0.811, 95%CI: 0.424-1.548, P = 0.525, I^2 = 69.3%). Due to the small number of included studies, robustness of results could not be determined by sensitivity analysis.

Supplementary Table 1. The PRISMA checklist.

Section and Topic	Item #	Checklist item	Location where item is reported
**TITLE**			
Title	1	Identify the report as a systematic review.	Title
**ABSTRACT**			
Abstract	2	See the PRISMA 2020 for Abstracts checklist.	Abstract
**INTRODUCTION**			
Rationale	3	Describe the rationale for the review in the context of existing knowledge.	Introduction
Objectives	4	Provide an explicit statement of the objective(s) or question(s) the review addresses.	Introduction
**METHODS**			
Eligibility criteria	5	Specify the inclusion and exclusion criteria for the review and how studies were grouped for the syntheses.	Inclusion and exclusion criteria
Information sources	6	Specify all databases, registers, websites, organisations, reference lists and other sources searched or consulted to identify studies. Specify the date when each source was last searched or consulted.	Search strategy
Search strategy	7	Present the full search strategies for all databases, registers and websites, including any filters and limits used.	Search strategy
Selection process	8	Specify the methods used to decide whether a study met the inclusion criteria of the review, including how many reviewers screened each record and each report retrieved, whether they worked independently, and if applicable, details of automation tools used in the process.	Data extraction and risk of bias assessment
Data collection process	9	Specify the methods used to collect data from reports, including how many reviewers collected data from each report, whether they worked independently, any processes for obtaining or confirming data from study investigators, and if applicable, details of automation tools used in the process.	Data extraction and risk of bias assessment
Data items	10a	List and define all outcomes for which data were sought. Specify whether all results that were compatible with each outcome domain in each study were sought (e.g. for all measures, time points, analyses), and if not, the methods used to decide which results to collect.	Inclusion and exclusion criteria
	10b	List and define all other variables for which data were sought (e.g. participant and intervention characteristics, funding sources). Describe any assumptions made about any missing or unclear information.	Inclusion and exclusion criteria

Section and Topic	Item #	Checklist item	Location where item is reported
Study risk of bias assessment	11	Specify the methods used to assess risk of bias in the included studies, including details of the tool(s) used, how many reviewers assessed each study and whether they worked independently, and if applicable, details of automation tools used in the process.	Data extraction and risk of bias assessment
Effect measures	12	Specify for each outcome the effect measure(s) (e.g. risk ratio, mean difference) used in the synthesis or presentation of results.	Statistical methods
Synthesis methods	13a	Describe the processes used to decide which studies were eligible for each synthesis (e.g. tabulating the study intervention characteristics and comparing against the planned groups for each synthesis (item #5)).	Inclusion and exclusion criteria
	13b	Describe any methods required to prepare the data for presentation or synthesis, such as handling of missing summary statistics, or data conversions.	Statistical methods
	13c	Describe any methods used to tabulate or visually display results of individual studies and syntheses.	Statistical methods
	13d	Describe any methods used to synthesize results and provide a rationale for the choice(s). If meta-analysis was performed, describe the model(s), method(s) to identify the presence and extent of statistical heterogeneity, and software package(s) used.	Statistical methods
	13e	Describe any methods used to explore possible causes of heterogeneity among study results (e.g. subgroup analysis, meta-regression).	Statistical methods
	13f	Describe any sensitivity analyses conducted to assess robustness of the synthesized results.	Sensitivity Analyses, eTable 5-7
Reporting bias assessment	14	Describe any methods used to assess risk of bias due to missing results in a synthesis (arising from reporting biases).	Data extraction and risk of bias assessment
Certainty assessment	15	Describe any methods used to assess certainty (or confidence) in the body of evidence for an outcome.	Data extraction and risk of bias assessment
**RESULTS**			
Study selection	16a	Describe the results of the search and selection process, from the number of records identified in the search to the number of studies included in the review, ideally using a flow diagram.	Study identification and characteristics, Figure 1

Section and Topic	Item #	Checklist item	Location where item is reported
	16b	Cite studies that might appear to meet the inclusion criteria, but which were excluded, and explain why they were excluded.	Study identification and characteristics
Study characteristics	17	Cite each included study and present its characteristics.	Study identification and characteristics
Risk of bias in studies	18	Present assessments of risk of bias for each included study.	Risk of bias, eTable 2-4
Results of individual studies	19	For all outcomes, present, for each study: (a) summary statistics for each group (where appropriate) and (b) an effect estimate and its precision (e.g. confidence/credible interval), ideally using structured tables or plots.	Section 3.5-3.9, Figure 2-5
Results of syntheses	20a	For each synthesis, briefly summarise the characteristics and risk of bias among contributing studies.	Risk of bias
	20b	Present results of all statistical syntheses conducted. If meta-analysis was done, present for each the summary estimate and its precision (e.g. confidence/credible interval) and measures of statistical heterogeneity. If comparing groups, describe the direction of the effect.	Section 3.5-3.9, Figure 2-5, eFigure 4-9
	20c	Present results of all investigations of possible causes of heterogeneity among study results.	Discussion
	20d	Present results of all sensitivity analyses conducted to assess the robustness of the synthesized results.	Section 3.5-3.9, Figure 2-5
Reporting biases	21	Present assessments of risk of bias due to missing results (arising from reporting biases) for each synthesis assessed.	eTable 2-4
Certainty of evidence	22	Present assessments of certainty (or confidence) in the body of evidence for each outcome assessed.	eTable 2-4
**DISCUSSION**			
Discussion	23a	Provide a general interpretation of the results in the context of other evidence.	Discussion
	23b	Discuss any limitations of the evidence included in the review.	Strengths and limitations
	23c	Discuss any limitations of the review processes used.	Strengths and limitations
	23d	Discuss implications of the results for practice, policy, and future research.	Strengths and limitations
**OTHER INFORMATION**			

Section and Topic	Item #	Checklist item	Location where item is reported
Registration and protocol	24a	Provide registration information for the review, including register name and registration number, or state that the review was not registered.	Search strategy
	24b	Indicate where the review protocol can be accessed, or state that a protocol was not prepared.	Search strategy
	24c	Describe and explain any amendments to information provided at registration or in the protocol.	Search strategy
Support	25	Describe sources of financial or non-financial support for the review, and the role of the funders or sponsors in the review.	Funding
Competing interests	26	Declare any competing interests of review authors.	Competing interests
Availability of data, code and other materials	27	Report which of the following are publicly available and where they can be found: template data collection forms; data extracted from included studies; data used for all analyses; analytic code; any other materials used in the review.	Availability of data and materials

From: Page MJ, McKenzie JE, Bossuyt PM, Boutron I, Hoffmann TC, Mulrow CD, et al. The PRISMA 2020 statement: an updated guideline for reporting systematic reviews. BMJ 2021;372:n71. doi: 10.1136/bmj.n71

For more information, visit: http://www.prisma-statement.org/

Supplementary Table 2. Results of the egger test for meta-analysis of patients with AF combined with CKD.

Outcome	No. of included studies	P value
Stroke or systemic embolism	7	0.288
Hemorrhagic stroke	4	0.915
Non-hemorrhagic stroke	7	0.001
VTE	5	0.552
All-cause death	6	0.509
Death from cardiac cause	3	0.566
Acute coronary syndrome	3	0.155
Major bleeding	8	0.388
Major or CRNM bleeding	3	0.265
Life-threatening bleeding	3	0.781
Intracranial bleeding	4	0.360
Gastrointestinal bleeding	4	0.419
Minor bleeding	3	0.666

CRNM bleeding, clinically relevant non-major bleeding.

Supplementary Table 3. Results of the egger test for meta-analysis of patients with VTE.

Outcome	No. of included studies	P value
Recurrent VTE or VTE-related death	5	0.435
Recurrent VTE	2	/
Major bleeding	3	0.719
Major bleeding or CRNM	3	0.816

VTE, venous thromboembolism; CRNM bleeding, clinically relevant non-major bleeding.

Supplementary Table 4. Results of the egger test for meta-analysis of patients with AF combined with severe CKD.

Outcome	No. of included studies	P value
Stroke or systemic embolism	3	0.795
Hemorrhagic stroke	2	/
Non-hemorrhagic stroke	3	0.388
Systemic embolism	2	/
All-cause death	4	0.583
Death from cardiac cause	3	0.886
Acute coronary syndrome	3	0.139
Major bleeding	4	0.833
Major or CRNM bleeding	2	/
Life-threatening bleeding	2	/
Intracranial bleeding	2	/
Gastrointestinal bleeding	2	/
Minor bleeding	2	/

Supplementary Table 5. Sensitivity analysis of studies included in the meta-analysis of patients with AF combined with CKD.

Outcome	Study omitted	Estimate	95% CI
Major bleeding	ARISTOTLE (2011)	0.62156069	0.43002313-0.89841133
	Vriese (2021)	0.60490888	0.43306264-0.8449465
	RENAL-AF (2022)	0.57333171	0.4131622- 0.79559368
	RE-LY (2013)	0.54251486	0.46337667-0.63516873
	AF-TIMI 48 Trial (2016)	0.60156846	0.40246665-0.8991667
	Chashkina (2020)	0.61427581	0.44613203-0.84579164
	Kumar (2024)	0.64787197	0.4735699- 0.88632768
	Chandra (2023)	0.62262696	0.44244269-0.87619108

Life-threatening bleeding	Vriese (2021)	0.75301629	0.39293584-1.4430689
	RENAL-AF (2022)	0.5835095	0.25676766-1.3260367
	RE-LY (2013)	0.3961035	0.20953792-0.74878091
	AF-TIMI 48 Trial (2016)	0.59117854	0.20549276-1.7007509
Minor bleeding	Vriese (2021)	0.70346123	0.54248518-0.9122051
	AF-TIMI 48 Trial (2016)	0.79936856	0.42014566-1.5208776
	Chashkina (2020)	0.8709963	0.6232748-1.2171751

95% CI: 95% confidence interval; CRNM bleeding, clinically relevant non-major bleeding.

Supplementary Table 6. Sensitivity analysis of studies included in the meta-analysis of patients with VTE combined with CKD.

Outcome	Study omitted	Estimate	95% CI
Major bleeding	AMPLIFY(2013)	0.54771352	0.09898368-3.0307028
	RE-COVERI/ II (2017)	0.36317465	0.16112587-.81858873
	EINSTEIN(2010)	0.79549742	0.32535404-1.9450078

95% CI: 95% confidence interval; VTE, venous thromboembolism; CRNM bleeding, clinically relevant non-major bleeding.

Supplementary Table 7. Sensitivity analysis of studies included in the meta-analysis of patients with AF combined with severe CKD.

Outcome	Study omitted	Estimate	95% CI
Stroke or systemic embolism	ARISTOTLE (2011)	3.5318339	0.31533697-39.557209
	Vriese (2021)	0.63731587	0.26526922-1.5311673
	RENAL-AF (2022)	2.4946027	0.14736916-42.227573

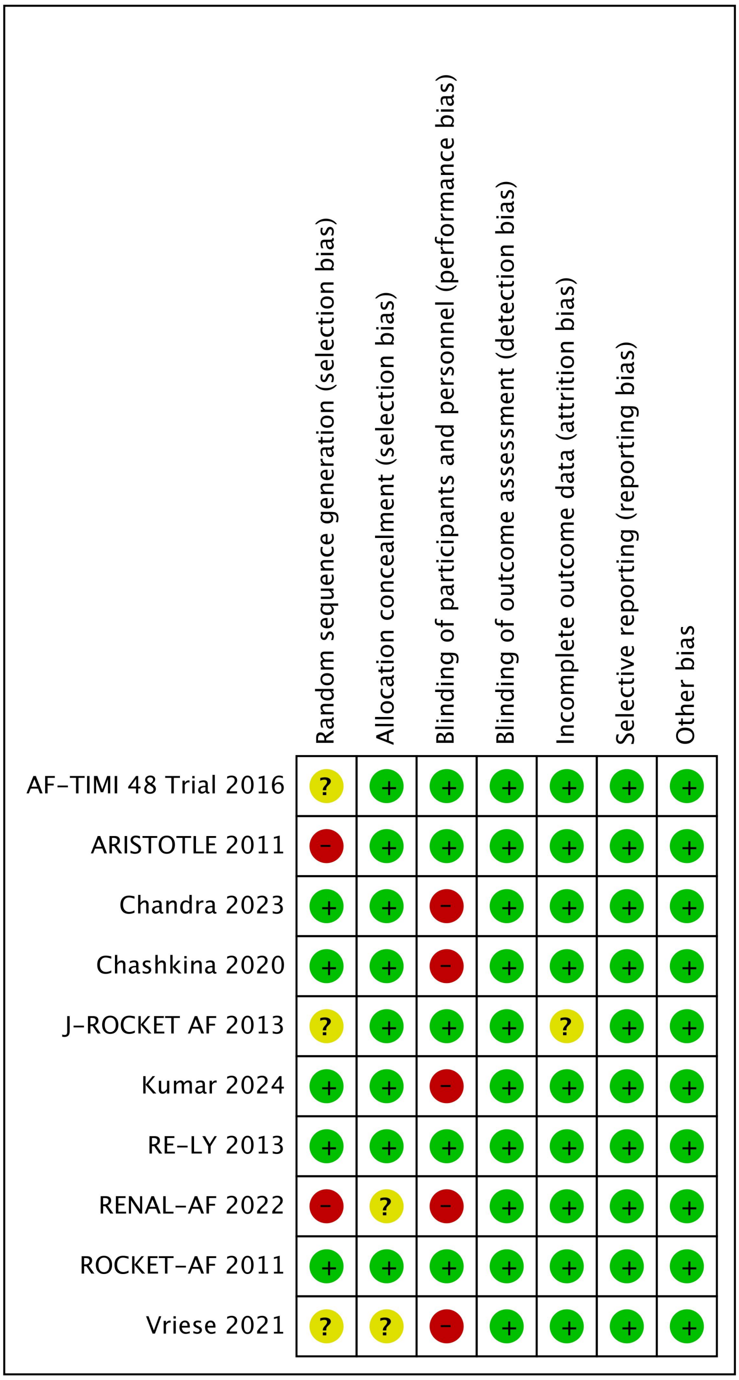
Random sequence generation (selection bias)
Allocation concealment (selection bias)
Blinding of participants and personnel (performance bias)
Blinding of outcome assessment (detection bias)
Incomplete outcome data (attrition bias)
Selective reporting (reporting bias)
Other bias
AF-TIMI 48 Trial 2016
ARISTOTLE 2011
Chandra 2023
Chashkina 2020
J-ROCKET AF 2013
Kumar 2024
RE-LY 2013
RENAL-AF 2022
ROCKET-AF 2011
Vriese 2021

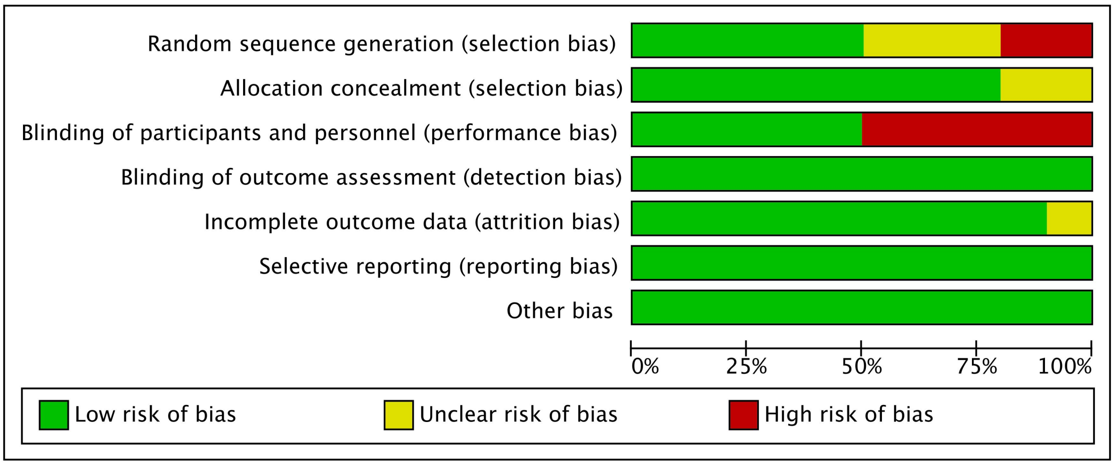
Random sequence generation (selection bias)
Allocation concealment (selection bias)
Blinding of participants and personnel (performance bias)
Blinding of outcome assessment (detection bias)
Incomplete outcome data (attrition bias)
Selective reporting (reporting bias)
Other bias
0%
25%
50%
75%
100%
Low risk of bias
Unclear risk of bias
High risk of bias

Supplementary Figure 1. Summary of risk of bias assessments for trials including Patients with CKD and AF.

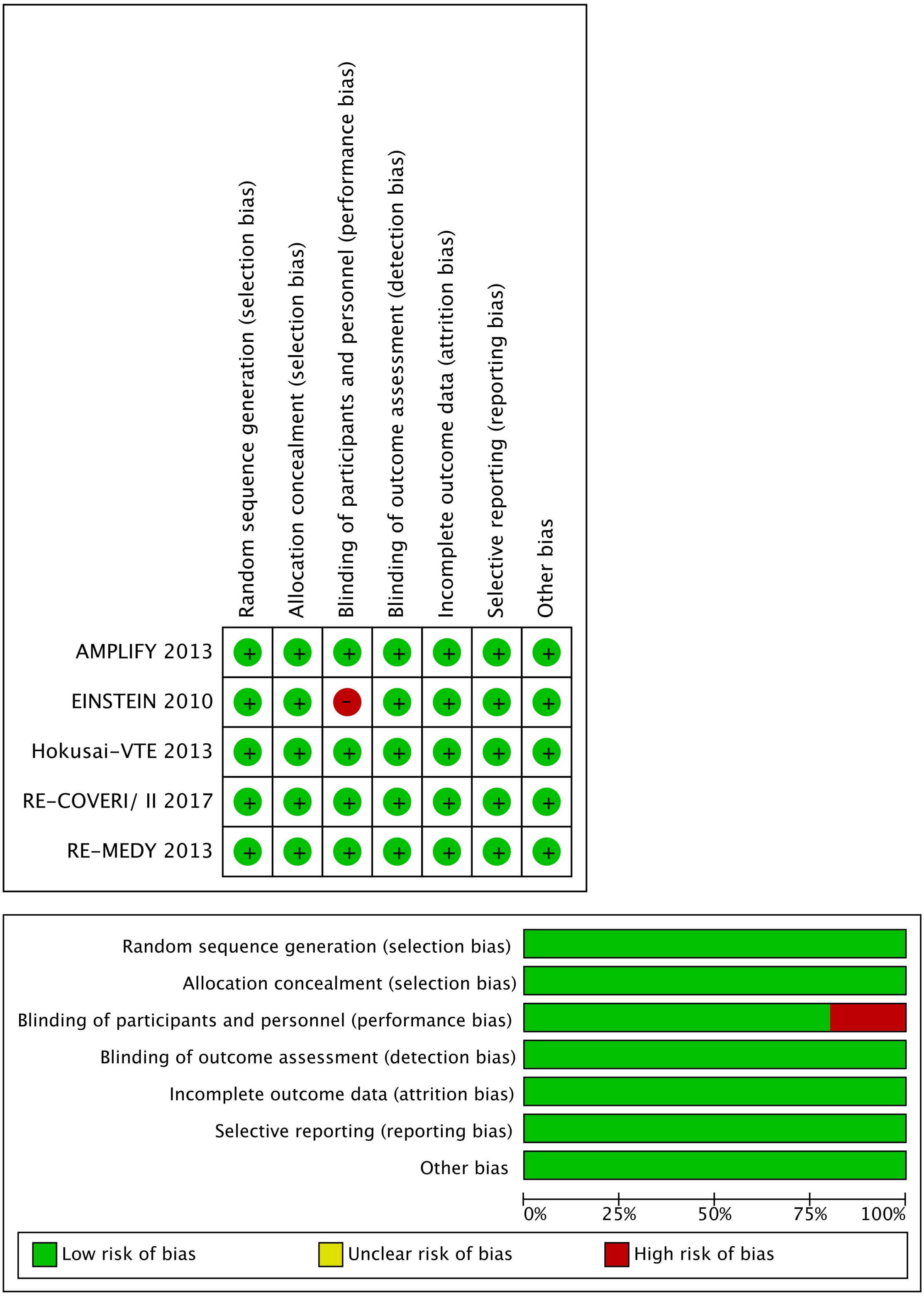

Supplementary Figure 2. Summary of risk of bias assessments for trials including Patients with CKD and VTE

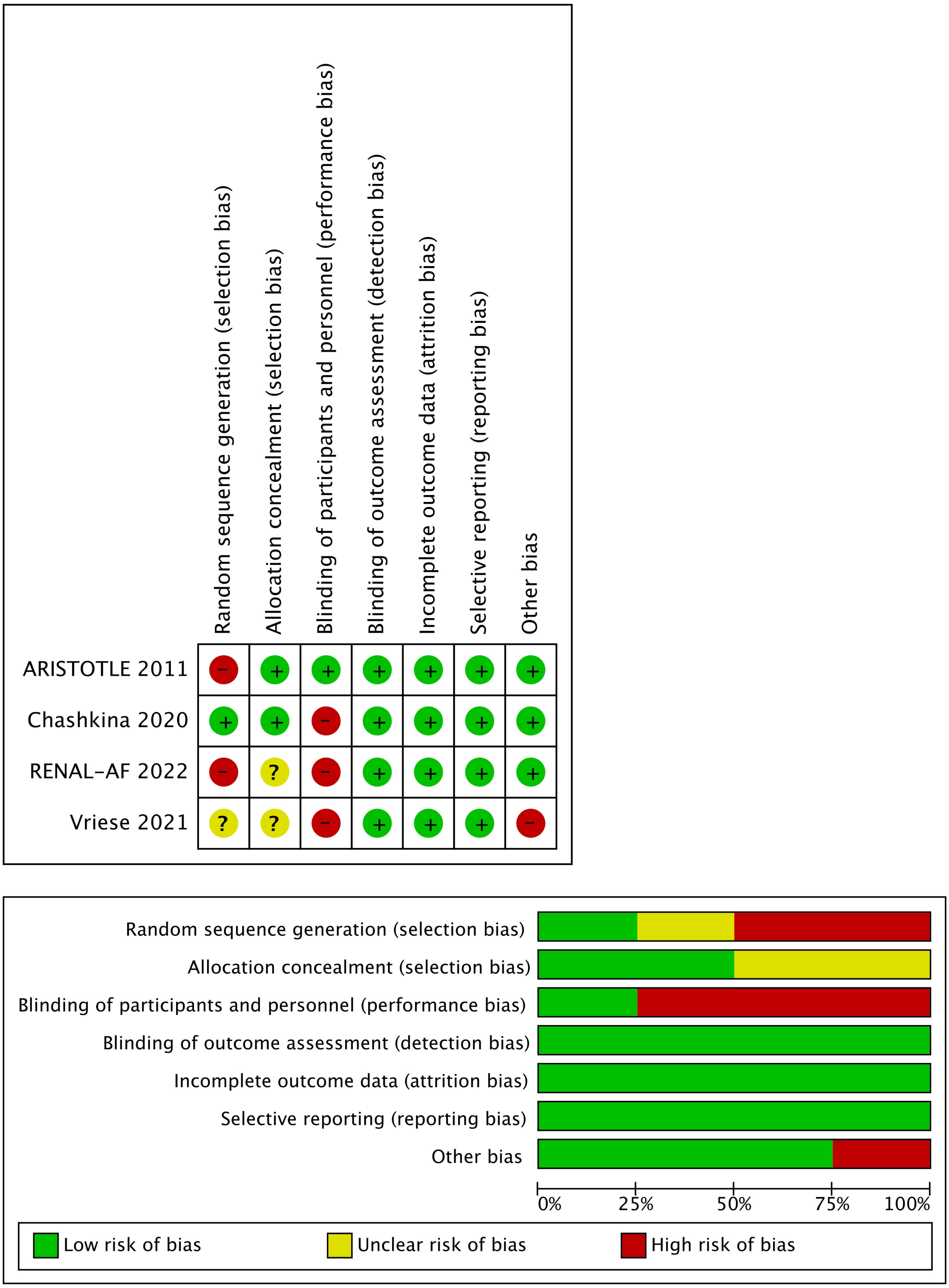

Supplementary Figure 3. Summary of risk of bias assessments for trials including Patients with severe CKD and AF.

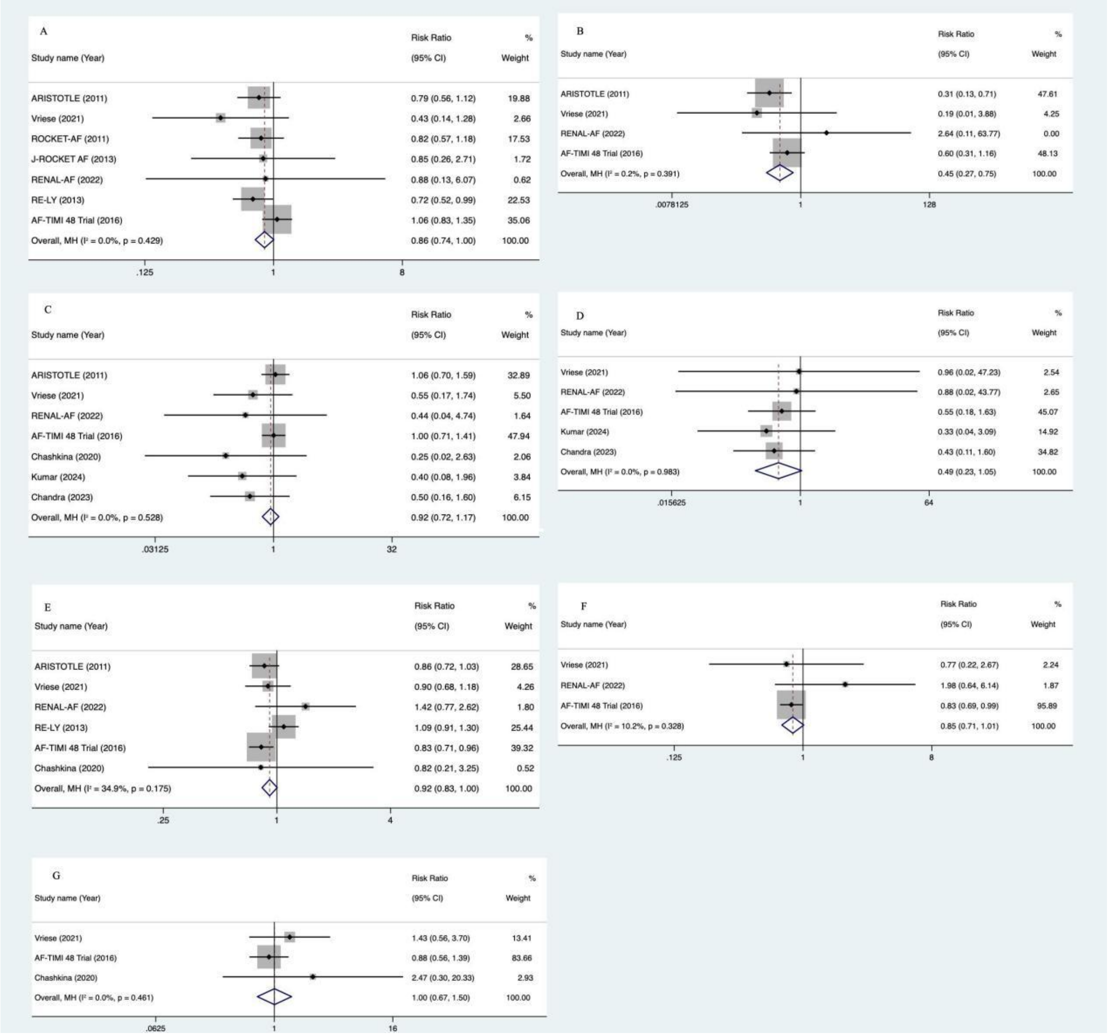

Supplementary Figure 4 Forest plot of effectiveness outcomes in patients with atrial fibrillation combined with CKD: stroke or systemic embolism (A), hemorrhagic stroke (B), non-hemorrhagic stroke (C), VTE (D), all-cause death (E), death from cardiac cause (F), and acute coronary syndrome (G)

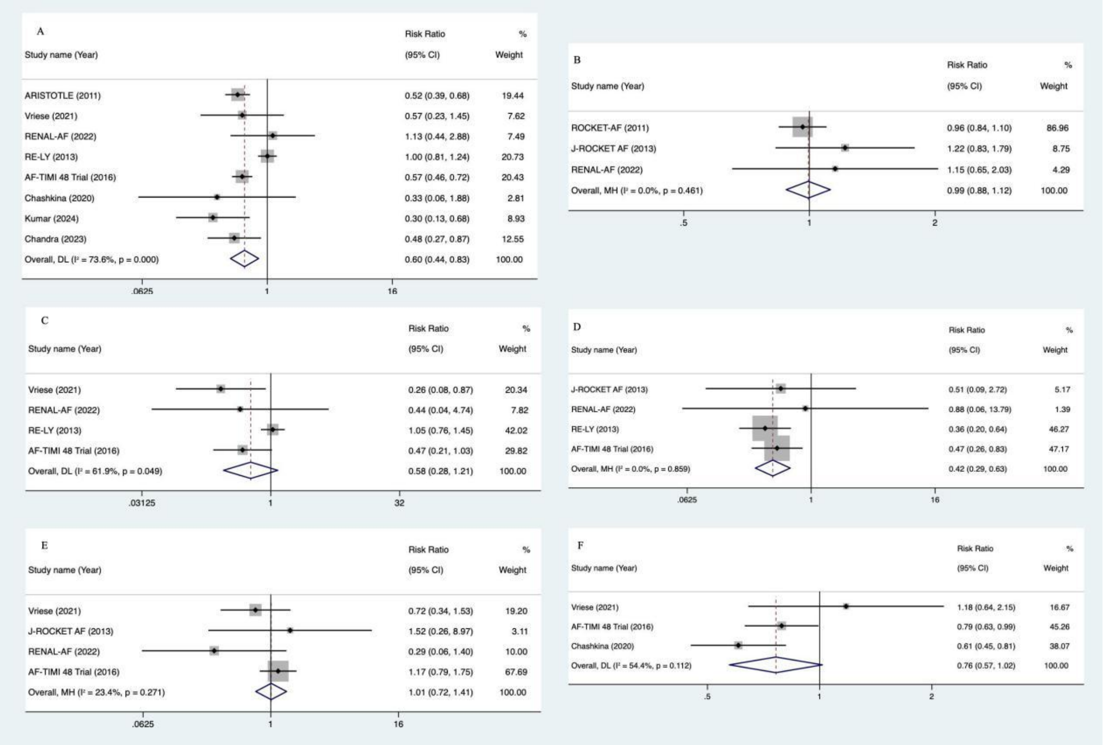

Supplementary Figure 5 Forest plot of safety outcomes in patients with atrial fibrillation combined with CKD: major bleeding (A), major or CRNM bleeding (B), life-threatening bleeding (C), intracranial bleeding (D), gastrointestinal bleeding (E), minor bleeding (F)

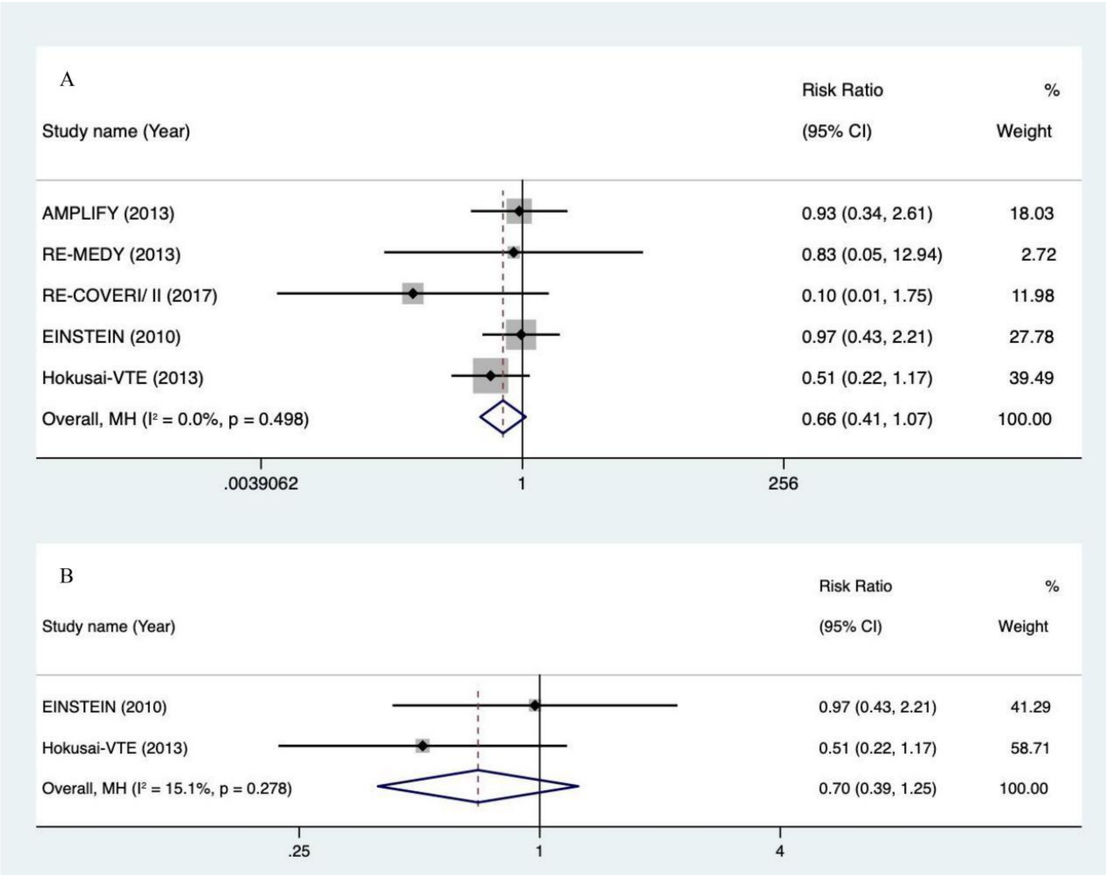

Supplementary Figure 6 Forest plot of effectiveness outcomes in patients with VTE combined with CKD: recurrent VTE or VTE-related deaths (A) and recurrent VTE (B)

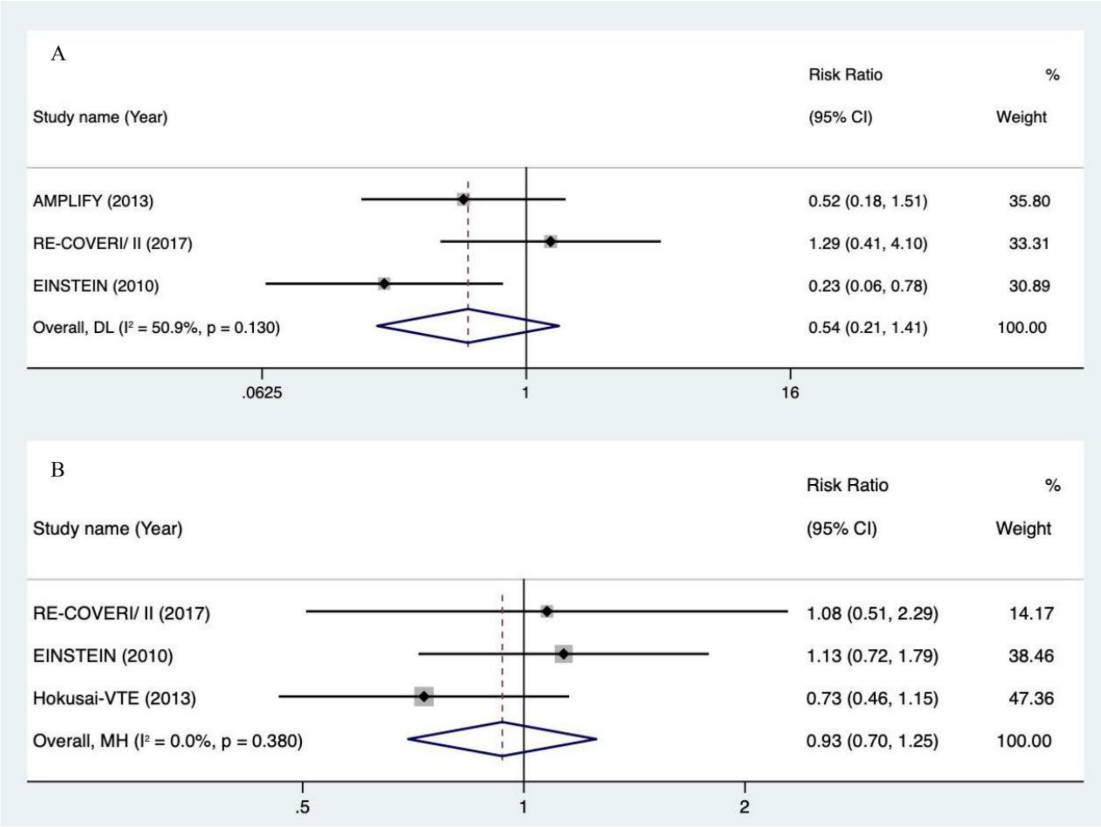

Supplementary Figure 7 Forest plot of safety outcomes in patients with VTE combined with CKD: major bleeding (A) and major or CRNM bleeding (B)

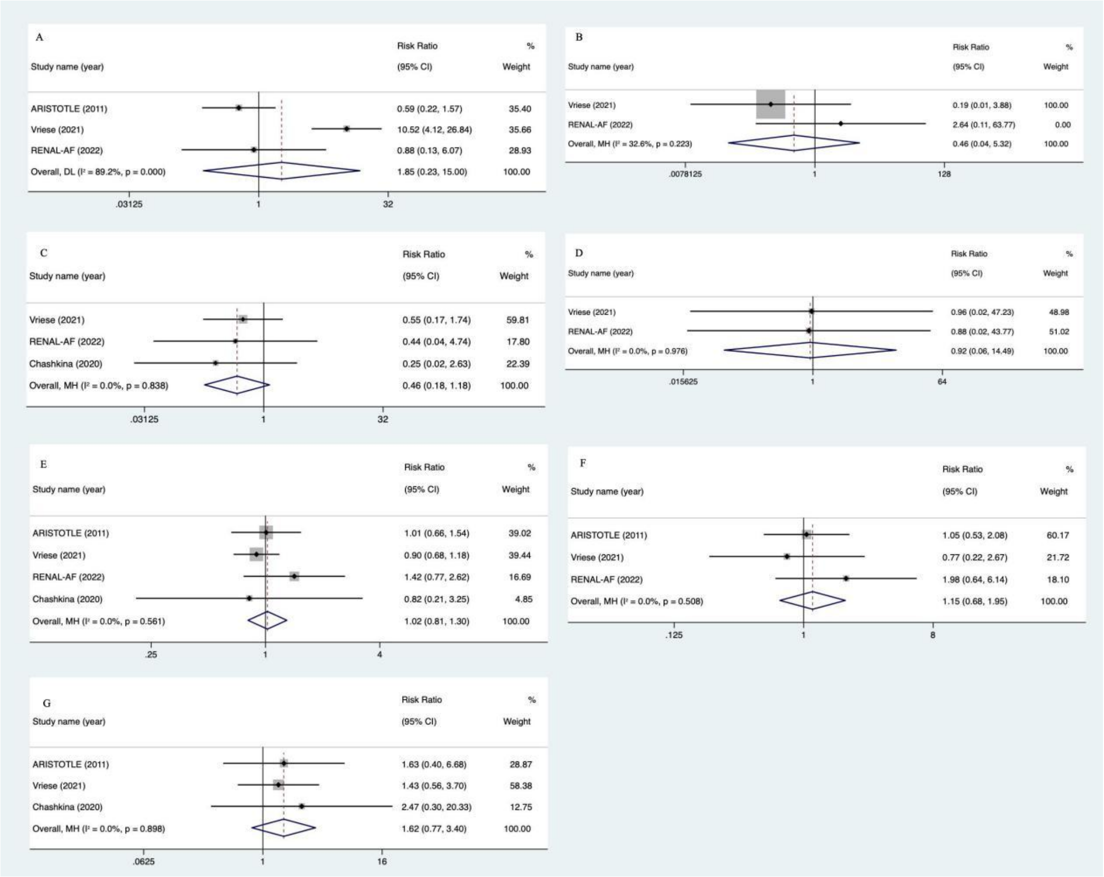

Supplementary Figure 8 Forest plot of effectiveness outcomes in patients with atrial fibrillation combined with severe CKD: stroke or systemic embolism (A), hemorrhagic stroke (B), non-hemorrhagic stroke (C), VTE (D), all-cause death (E), death from cardiac cause (F), and acute coronary syndrome (G)

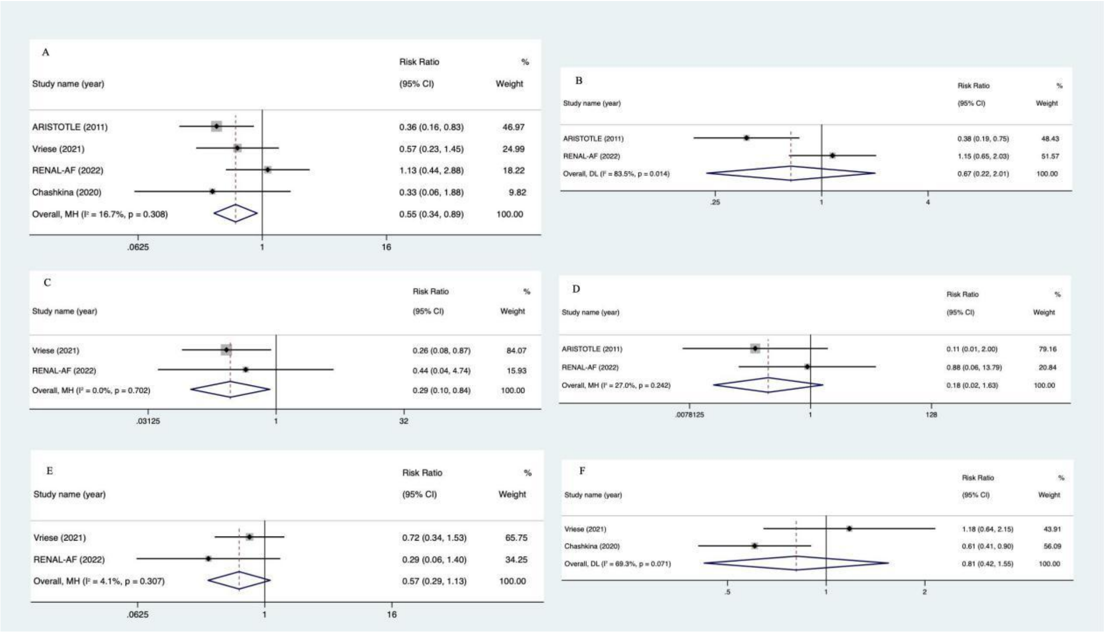

Supplementary Figure 9 Forest plot of safety outcomes in patients with atrial fibrillation combined with severe CKD: major bleeding (A), major or CRNM bleeding (B), life-threatening bleeding (C), intracranial bleeding (D), gastrointestinal bleeding (E), minor bleeding (F)

1. De Vriese, A.S., et al., *Safety and Efficacy of Vitamin K Antagonists versus Rivaroxaban in Hemodialysis Patients with Atrial Fibrillation: A Multicenter Randomized Controlled Trial.* J Am Soc Nephrol, 2021. **32**(6): p. 1474-1483.
